# Feasibility and Acceptability of Using a Mobile Phone App for Characterizing Auditory Verbal Hallucinations in Adolescents With Early-Onset Psychosis: Exploratory Study

**DOI:** 10.2196/13882

**Published:** 2019-05-14

**Authors:** Runar Elle Smelror, Josef Johann Bless, Kenneth Hugdahl, Ingrid Agartz

**Affiliations:** 1 Department of Psychiatric Research Diakonhjemmet Hospital Oslo Norway; 2 NORMENT Center of Excellence Institute of Clinical Medicine University of Oslo Oslo Norway; 3 Department of Biological and Medical Psychology University of Bergen Bergen Norway; 4 NORMENT Center of Excellence Haukeland University Hospital Bergen Norway; 5 Division of Psychiatry Haukeland University Hospital Bergen Norway; 6 Centre for Psychiatric Research Department of Clinical Neuroscience Karolinska Institutet Stockholm Sweden

**Keywords:** experience sampling method, ecological momentary assessment, schizophrenia, mHealth, health care technology

## Abstract

**Background:**

Auditory verbal hallucinations (AVH) are the most frequent symptom in early-onset psychosis (EOP) and a risk factor for increased suicide attempts in adolescents. Increased knowledge of AVH characteristics can lead to better prediction of risk and precision of diagnosis and help identify individuals with AVH who need care. As 98% of Norwegian adolescents aged 12 to 16 years own a mobile phone, the use of mobile phone apps in symptom assessment and patient communication is a promising new tool. However, when introducing new technology to patients, their subjective experiences are crucial in identifying risks, further development, and potential integration into clinical care.

**Objective:**

The objective was to explore the feasibility and acceptability of a newly developed mobile phone app in adolescents with EOP by examining compliance with the app and user experiences. Indication of validity was explored by examining associations between AVH dimensions, which were correlated and analyzed.

**Methods:**

Three adolescents with EOP and active AVH were enrolled. Real-time AVH were logged on an iPod touch using the experience sampling method (ESM), for seven or more consecutive days. The app included five dimensions of AVH characteristics and was programmed with five daily notifications. Feasibility and acceptability were examined using the mean response rate of data sampling and by interviewing the participants. Validity was assessed by examining associations between the AVH dimensions using nonparametric correlation analysis and by visual inspection of temporal fluctuations of the AVH dimensions.

**Results:**

One participant was excluded from the statistical analyses but completed the interview and was included in the examination of acceptability. The sampling period of the two participants was mean 12 (SD 6) days with overall completed sampling rate of 74% (SD 30%), indicating adequate to high compliance with the procedure. The user experiences from the interviews clustered into four categories: (1) increased awareness, (2) personal privacy, (3) design and procedure, and (4) usefulness and clinical care. One participant experienced more commenting voices during the sampling period, and all three participants had concerns regarding personal privacy when using electronic devices in symptom assessment. The AVH dimensions of content, control, and influence showed moderate to strong significant correlations with all dimensions (*P*<.001). Days of data sampling showed weak to moderate correlations with localization (*P*<.001) and influence (*P*=.03). Visual inspection indicated that the app was able to capture fluctuations within and across days for all AVH dimensions.

**Conclusions:**

This study demonstrates the value of including patients’ experiences in the development and pilot-testing of new technology. Based on the small sample size, the use of mobile phones with ESM seems feasible for patients with EOP, but the acceptability of using apps should be considered. Further investigation with larger samples is warranted before definitive conclusions are made.

## Introduction

Early-onset psychosis (EOP) is psychotic disorders with age of onset before 18 years of age. EOP is often characterized by a chronic illness course with impaired social and daily functioning [1,2]. The most frequent symptom in EOP is auditory verbal hallucinations (AVH), which is found in 82% of patients [3]. Although AVH are relatively common among adolescents in the general population (prevalence ranging from 7.3% to 12.4%) [4-6], 34% of adolescents with AVH and psychopathology have had at least one suicide attempt compared to 13% of adolescents without AVH [7]. This emphasizes the need to further investigate AVH in youth to better identify individuals with need for care and learn more about the underlying mechanisms causing this phenomenon [8].

Assessing AVH using traditional retrospective measures, such as the Positive and Negative Syndrome Scale (PANSS) [9] or the Scale for the Assessment of Positive Symptoms (SAPS) [10], may lead to loss of symptom information due to recall errors and state-dependent biases [11-13]. Real-time monitoring of symptoms increases the ecological validity and enables researchers to gather new and different information from patients [12,14,15]. The experience sampling method (ESM) [16] was originally based on self-reports (such as diary entries) to monitor real-time experiences and symptom frequency daily. Therefore, ESM would seem well-suited for use with mobile phone apps to assess and monitor psychopathology and symptom characteristics in patients with schizophrenia [15,17-20], including AVH [21]. In an ESM study of adults with schizophrenia spectrum and affective disorders, Delespaul and colleagues [22] found that AVH had higher intensity than visual hallucinations, and that anxiety was the strongest predictor of hallucination intensity compared to other mental states. They also found that anticipatory anxiety was present before the start of AVH, but not for visual hallucinations.

In Norway, 98% of adolescents aged 12 to 16 years own a mobile phone [23], and the Norwegian Health Authority suggests using mobile phones in communication with youth with EOP to support school attendance [24]. The use of mobile phone apps for symptom assessment in adolescents and young adults with psychotic disorders is feasible over longer time periods [25,26]. There are at least 11 mobile phone apps that have been developed for schizophrenia [20,27-33]. However, most of the apps focus on self-management, in which symptoms are a small part [21]. Increased knowledge of AVH characteristics, such as localization (external/internal), cognitive control (uncontrollable/controllable), distress (negative/positive), and fine-grained temporal patterns of frequency and fluctuations, can lead to better quantification of symptom dynamics over time and contribute to new treatment opportunities. This could come about through improving the prediction of risk and precision of diagnosis, identifying needs for services, enabling measurement-based care, and increasing autonomy and independence in patients [34-36]. When introducing new technology to vulnerable groups, such as EOP patients, the risks need to be considered against potential benefits [35]. Thus, patients’ subjective experiences are crucial in further development and potential integration of new technology into clinical care [37,38], which was a focus of this study.

We piloted a newly developed mobile phone app designed to monitor AVH characteristics at the moment they occur in adolescents with EOP. It is of particular interest to investigate these phenomena in adolescents because this life period is a challenging maturation process for neurological and psychological development [39,40], and because they are less influenced by confounding factors (eg, long-term medication use, lifestyle-related illnesses, substance use). The objective was to explore the feasibility and acceptability of the app by examining compliance with the sampling procedure and the patients’ subjective experiences. In addition, validity was explored by analyzing associations between the five AVH dimensions included in the app. Data collected with the app have also been analyzed in a larger sample of patients, focusing on the statistical aspects of the AVH dimensions (JB and KH, unpublished data, 2019). Those results are not presented in this study.

## Methods

### Participants

The study was approved by the South East Regional Committee for Medical and Health Research Ethics, Oslo, Norway, and conducted in accordance with the Helsinki Declaration. Four adolescents with EOP and active AVH, already included in the larger ongoing Thematically Organized Psychosis Study for Youth (Youth-TOP) from 2016 to 2018, were invited to participate in this pilot study. One patient refused to participate for unknown reasons, resulting in a total of three enrolled participants. Of the three, one used the app in a psychiatric unit and two used it in their home environment.

The participants were recruited from adolescent psychiatric inpatient units and outpatient clinics in the Oslo region. The inclusion criteria were (1) nonaffective EOP (schizophrenia, schizoaffective disorder, schizophreniform disorder, psychotic disorder not otherwise specified, brief psychotic disorder) according to the *Diagnostic and Statistical Manual of Mental Disorders* (Fourth Edition); (2) having experienced AVH during the past week at the time of inclusion, defined as scoring 4 or higher on item P3 of the PANSS [9]; (3) written informed consent obtained from participants, parents, or guardians (if the participant was younger than 16 years); and (4) language abilities to complete the interviews and self-rating questionnaires. Exclusion criteria were (1) general intelligence quotient (IQ) less than 70, (2) previous moderate/severe head injury, (3) diagnosis of substance-induced psychosis, (4) organic brain disease, and (5) noncompliance to the sampling procedure (ie, insufficient amount of self-assessments in the app, not using the app for a minimum of seven consecutive days). The participants received monetary compensation of 500 Norwegian kroner (approximately €50) after completing the sampling period. They were informed that participation was voluntary, it would not influence their clinical care, and they could stop using the app at any time and still get the monetary compensation.

### Measures

Diagnoses were confirmed using the Schedule for Affective Disorders and Schizophrenia for School-Age Children-Present and Lifetime version [41]. Global functioning was assessed using the Children’s Global Assessment Scale (CGAS) [42]. IQ was assessed using the Wechsler Abbreviated Scale of Intelligence [43]. Current psychopathology, including degree of hallucinatory experiences, and individual beliefs about AVH were assessed before and after the app sampling period using the PANSS and the Beliefs About Voices Questionnaire-Revised version (BAVQ-R) [44,45]. The BAVQ-R includes three subscales regarding beliefs about voices: malevolence (“bad voices”), benevolence (“good voices”), and omnipotence (“powerful voices”) and two subscales regarding emotional and behavioral reactions to voices: resistance and engagement. The participants’ subjective experiences were collected using a semistructured user-experience interview developed in-house by us.

### Overview of the Mobile Phone App

**Figure 1:**
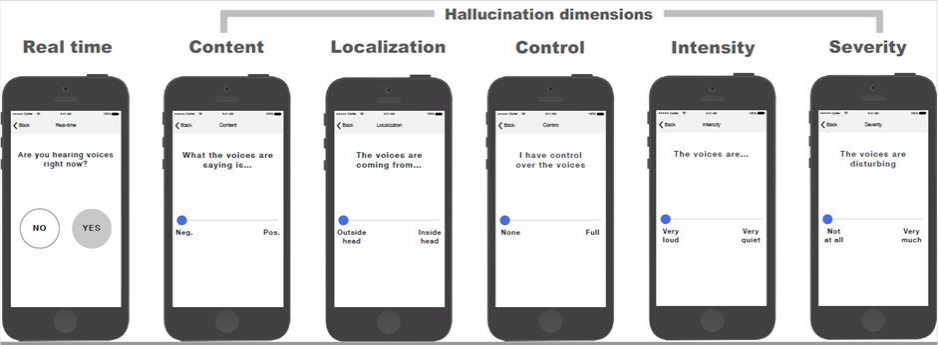
How the app is used by the participants. In the first screen the participant chooses yes or no to whether they hear voices right now. If they answer “yes,” the five following screens with the visual analogue scales for the five auditory verbal hallucinations (AVH) dimensions appear. Each screen represents the following AVH characteristic: content (emotional), localization (perceptual), control (cognitive), intensity (intensity), and influence (influence). Previously presented on a poster at the International Congress on Schizophrenia Research, March 24-28, 2017; San Diego, CA, with the abstract by Bless et al [46]. iPhone frames adopted from a design by Todd Zaki Warfel.

The mobile phone app was originally developed by coauthors KH and JB at the University of Bergen, Norway (see [46]). It was programmed in Xcode 4.2 (Apple Inc, Cupertino, CA, US), which makes it compatible with iOS devices such as the iPod touch. The selected AVH dimensions in the app were based on relevant research (see Johns and colleagues [47]). We received input from one service user during the development. The app includes five visual analog scales (VAS), each representing a separate AVH dimension: cognitive control (no control/full control of voices), emotionality of content (negative/positive voices), perceptual localization (voices perceived outside/inside head), intensity (voices are yelling/whispering), and influence (not at all/very disturbing voices). The outline of the app is illustrated in Figure 1. The prototype of the app was reviewed and approved by an adult user representative before pilot-testing with adolescents was initiated.

### Procedure

The participants sampled real-time AVH experiences on an iPod touch (Apple Inc, Cupertino, CA, US) provided by us, with a preinstalled version of the app. We chose to use iPods instead of mobile phones in the pilot study to guarantee the personal privacy of the participants. The iPods were not connected to the internet. The app was programmed with five daily notifications at pseudorandom time points from 10:00 to 22:00 (within five time intervals of 1.5 hours), in accordance with recommendations from Palmier-Claus and colleagues [48]. The participants were asked to respond to the five daily notifications as soon as possible after the beep, for a minimum of seven consecutive days. They were also informed that they could sample data in addition to the five times if they experienced AVH. Before and after completing the data sampling period the participants were asked to complete the BAVQ-R and a short version of the PANSS, covering the positive subscale. The sampling period began immediately after instructions of the app were given and the informed consent was collected. To facilitate compliance with the procedure, the first author (RES) contacted the participants once per week to ask about any difficulties using the app and offer support. The app file contained the date and time of the reports, and the VAS scores (ranging from 0 to 1) in chronological order. No personal user information was stored on the iPod. After completing the sampling period, user-experience interviews were conducted with each participant by author RES. To increase the face validity of the participant responses, alliance and trust were established before the interviews. Moreover, one of the participants’ parents was present during the interviews, and the participants were encouraged to report their subjective experiences of the app, either positive or negative.

### Data Analysis

Statistical analyses were performed using IBM SPSS Statistics for Windows version 25. Feasibility was examined using the mean response rate of the data sampling, calculated by dividing the total number of responses by days using the app multiplied by five (as five daily registrations indicated 100% compliance). Acceptability was examined by interviewing the participants about their subjective experiences of using the app. The participant experiences were written down. No voice or video recordings were used. Thus, the transcriptions of the participants’ subjective experiences are not verbatim quotes, but reproductions based on the written material. Indication of validity was assessed by examining associations between the AVH dimensions using two-tailed Spearman rho (ρ) nonparametric correlation analysis and visual investigation of the fluctuations of the AVH dimensions. This was done by making a graph with the mean symptom scores and days of sampling, defined as the number of data entries. Different time intervals between each data entry were not taken into consideration (ie, the time period between the first and second data entry might have differed from the second and third entry).

## Results

### Participants

The demographics of the three participants were mean age 17.7 (SD 1.6) years, mean age of onset for psychosis 14.9 (SD 0.7) years, mean duration of untreated psychosis 32 (SD 22) weeks, mean illness length 2.8 (SD 1.8) years, mean IQ score 93.7 (SD 8.0), and mean CGAS score 36 (SD 4). Two of the three participants were taking atypical antipsychotic medications. One of the three was excluded from the statistical analyses due to noncompliance with the procedure. This participant did not sample real-time data for seven consecutive days and had some aggregated registrations (ie, five samplings at the same time during the afternoon/evening). According to the participant, the reason for noncompliance was partly due to having to bring two devices to daily activities. The participant did not differ from the remaining two regarding age, sex, global functioning, or symptom severity. Thus, the participant’s subjective experiences were included in the examination of acceptability. The average sampling period of the two remaining participants was mean 12 (SD 6) days with a mean response rate of 74% (SD 30%) indicating adequate to high compliance with the sampling procedure.

### Clinical Assessment

The participants had stable PANSS scores before and after the sampling period with an average positive sum score of 14 and a P3 score of 4. The malevolence scale on the BAVQ-R was reduced by 3 points, from 11.0 to 8.0. The omnipotence scale was similarly reduced by 1.6 points, from 13.3 to 11.7. The resistance scale was also reduced by 0.7 points, from 16.0 to 15.3, and the engagement scale was reduced by 2 points, from 6.0 to 4.0. The BAVQ scores thus showed a general improvement in AVH characteristics regarding malevolence and omnipotence during the sampling period.

### Acceptability

Mixed experiences of the app were reported when exploring the acceptability, clustered into four main categories: (1) increased awareness, (2) personal privacy, (3) design and procedure, and (4) usefulness and clinical care. Examples of the participant subjective reports are presented in Table 1.

**Table 1:** Illustrations of participant experiences after the sampling period (N=3).

Experience	Sample statements^a^
Increased awareness	I became aware at every registration. It was okay to become more aware. Nothing was negative.
	I was reminded of the voices. I usually try to avoid that. I got more commenting voices, saying ‘now he/she’s doing it again, why is he/she registering us?’ That’s why I haven’t used it so much.
Personal privacy	I was a bit worried of being watched when I used the app. I saw the camera in front of the iPod. I don’t like cameras.
	I have felt monitored when using the app, particularly in the beginning, because of the notifications. I liked that it was offline. I’m afraid of leaving traces on the internet.
	It’s not secure, you may get hacked.
Design and procedure	The app was short and easy to understand. It was a bit much of the same questions. I was watching the iPod all the time, not knowing when the next notification would come. I thought a lot about when the next notification would come.
	It was okay with five notifications per day. If the notifications came at set time points it could help reduce thoughts about being monitored. I liked that I could write notes at the end.
	The layout was nice; the visual scales and the order of the statements were nice. Maybe three registrations in one day instead.
Usefulness and clinical care	It wasn’t very useful. Might be helpful to others, I don’t know.
	Nice to register symptoms. It would be very nice to have in clinical care. When I’m feeling down it’s hard to answer questions. It’s okay to use the app if the voices are not in control, that they don’t have opinions about what you should answer. If the voices are in control you might give the wrong answer.
	It was useful to learn about the voices. It was difficult to remember to sample data toward the end. I forgot the iPod at home or in my backpack. I don’t want it as part of my treatment, I prefer to talk.

^a^The statements are illustrations of the participants’ subjective experiences, not quotes.

### Increased Awareness

Two participants reported becoming more aware of their AVH when using the app. One participant reported that the increased awareness was positive, whereas the other reported it as negative, resulting in more commenting voices. This was reported as the main reason for stopping using the app by the latter participant.

### Personal Privacy

The participants had concerns regarding personal privacy when using apps and technology as part of their symptom assessment. Although they knew that the iPod was offline during the sampling period, they reported having fears of being monitored through the camera or the notifications from the app. In general, they reported worries about leaving electronic traces on the internet and being hacked. All participants considered it positive that the iPod was not connected to the internet during the pilot testing. This may be an argument to use iPod-like devices rather than real mobile phones to reduce these kinds of worries. However, it should be noted that such worries and concerns may also be part of the illness, as increased suspiciousness of being “monitored” from the outside is a not uncommon symptom.

### Design and Procedure

The participants liked the design of the app, that it was short (completed the self-assessments in about 1 minute), easy to understand, and it had the option of writing notes at the end. One participant would have preferred having numbers on the VAS scales to make scoring easier. Two participants reported that five daily notifications were adequate, whereas one thought this was too much and suggested three as an alternative. Two participants reported discomfort from the random notifications, reporting thoughts of being monitored and concerns and preoccupations regarding the next notification. They suggested having notifications at set times. Furthermore, one participant reported that the repetitiveness of the self-assessments was a bit much when sampling data five times per day for one week, and suggested adding more varied self-assessments to the app. The reports were mixed, which points to the importance of considering more technical issues such as design when developing apps.

### Usefulness and Clinical Care

Two participants reported that it was nice to register symptoms and learn more about the voices. However, the same participants reported decreased motivation toward the end of the sampling period and when their mood was low. One participant reported that using technology in treatment could be a useful alternative to answer verbal questions, but that self-reports could be biased if the voices were in control and had opinions about what to report. This is not an argument against the use of apps because the same could apply using traditional self-reports. However, the statement indicates that personal contact is an important part of clinical care and that apps and technology potentially can be used to monitor remission and relapse as part of measurement-based care, as suggested by Insel [35]. Two participants did not want to include the app as part of their clinical care. All participants reported concerns about personal privacy regarding the use of technology in clinical care.

### Validity

**Figure 2:**
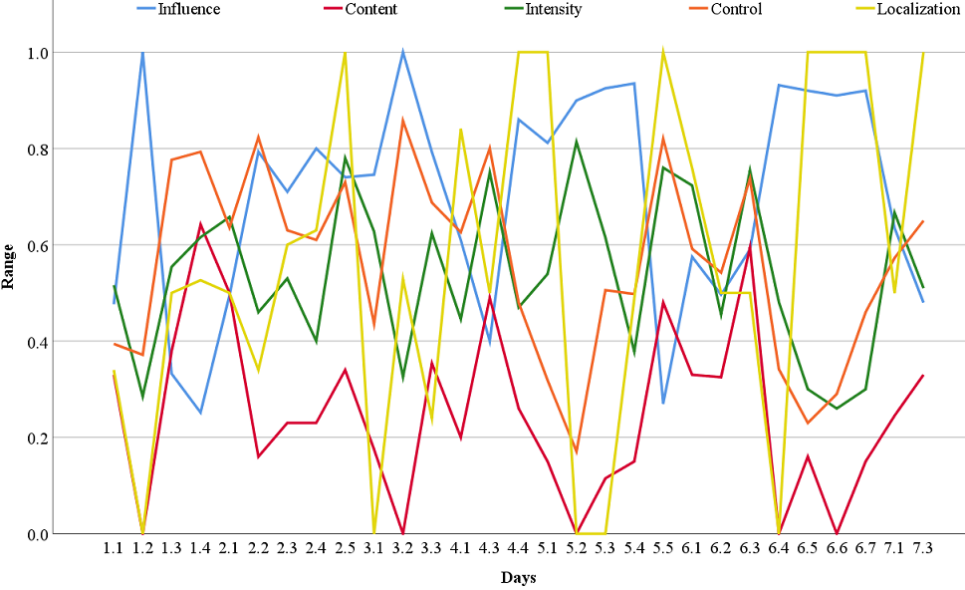
Mean scores of two participants for the five auditory verbal hallucination dimensions within and across 7 days of sampling.

Indications of validity were assessed by examining associations between the AVH dimensions and by examining the fluctuations of the AVH dimensions during 1 week of sampling. Table 2 shows correlations between the five AVH dimensions and days of sampling (ie, number of data entries). Content, control, and influence showed moderate (defined as ρ=±.5-.7 [49]) to strong (defined as ρ=±.7-.9) significant correlations with all dimensions: content and influence (ρ=–.80, *P*<.001), control (ρ=.73, *P*<.001), intensity (ρ=.43, *P*<.001), and localization (ρ=.50, *P*<.001); control and influence (ρ=–.77, *P*<.001), intensity (ρ=.48, *P*<.001), and localization (ρ=.41, *P*<.001); and influence and intensity (ρ=–.44, *P*<.001) and localization (ρ=–.56, *P*<.001). No significant correlation was found between intensity and localization. Days of data sampling showed weak (defined as ρ=±.3-.5) to moderate correlations with localization (ρ=.51, *P*<.001) and influence (ρ=–.25, *P*=.03), and nonsignificant correlations with content (ρ=.22, *P*=.06) and control (ρ=.21, *P*=.07). As shown in Figure 2, the app was able to capture the fluctuations of the five AVH dimensions within and across 7 days of sampling.

**Table 2:** Spearman rho (ρ) correlations for the participants completing the sampling period (N=2). Influence: not at all (0) to very disturbing (1); content: negative (0) to positive (1); intensity: yelling (0) to whispering (1); control: no control (0) to full control (1); localization: outside head (0) to inside head (1). All correlations were two-tailed.

AVH^a^ dimension	Content		Control		Influence		Intensity		Localization		Day	
	ρ	*P* value	ρ	*P* value	ρ	*P* value	ρ	*P* value	ρ	*P* value	ρ	*P* value
Content	1	―^b^	.73	<.001	–.80	<.001	.43	<.001	.50	<.001	.22	.06
Control	.73	<.001	1	―	–.77	<.001	.48	<.001	.41	<.001	.21	.07
Influence	–.80	<.001	–.77	<.001	1	―	–.44	<.001	–.56	<.001	–.25	.03
Intensity	.43	<.001	.48	<.001	–.44	<.001	1	―	.12	.30	.05	.64
Localization	.50	<.001	.41	<.001	–.56	<.001	.12	.30	1	―	.51	<.001
Day	.22	.06	.21	.07	–.25	.03	.05	.64	.51	<.001	1	―

^a^AVH: auditory verbal hallucinations.

^b^Not applicable.

## Discussion

The objective of this exploratory study was to examine the feasibility, acceptability, and indication of validity of a newly developed mobile phone app for auditory verbal hallucination characteristics in adolescents with EOP.

### Auditory Verbal Hallucination Fluctuations Captured by the Experience Sampling Method

Although the statistical analyses of this study only included two participants, the correlation analyses and the graph containing within and across day fluctuations indicate that the app is valid for use in adolescents with EOP. The content, control, and influence dimensions were significantly correlated with all the AVH dimensions, indicating that these characteristics are important among these patients. This finding is in accordance with a study in adults with AVH, showing that experiences of mostly negative, uncontrollable, and distressing voices were important characteristics in separating nonpsychotic individuals from individuals with psychotic disorders [34]. Furthermore, the presence of this pattern of AVH characteristics (ie, negative content associated with less control and more disturbing voices) required that the participants consistently scored the influence dimension in the opposite direction to the content and control dimensions on the VAS scales, which confirms intentional responses from the participants. This indicates that the AVH dimensions in the app demonstrate internal consistency and construct validity, although further investigations in larger samples must be conducted before conclusions can be made. We also found significant correlations between the negative content, lack of control and disturbing voices, and external localization, suggesting that the AVH were perceived as more outside their heads when they were negative, uncontrollable, or distressing. However, perceived localization of voices was not significantly different between nonpsychotic and psychotic patients, in which individuals with psychotic disorders to a similar extent perceived the voices coming from inside their heads [34].

### Increased Awareness

The user-experience interviews revealed that two participants experienced increased awareness of the AVH when using the app, highlighting the need for researchers to consider that the assessment process itself may exert an influence on the individual through reactivity effects [50]. Increased self-awareness is associated with increased negative affect, particularly in individuals with existing high negative affectivity [51,52]. In a mobile phone study of happiness, Conner and Reid [53] found a negative association between happiness and number of daily reports in participants with high negative affectivity, whereas a positive association was found in participants with low negative affectivity. The authors suggested that frequent data sampling increased the participants’ self-awareness, which in turn intensified the underlying emotional state (negative or positive) [53]. The increased awareness is in accordance with a previous study of adults with schizophrenia, showing increased preoccupation with thoughts when repeatedly assessing symptoms using a mobile phone [36]. In terms of metacognitive theory [54], this finding suggests that repetitive sampling of AVH increased the participants’ metacognitive level of cognitive self-consciousness (ie, preoccupation of thoughts; see also [55,56]). The findings can be interpreted that both participants in our study showed increased levels of cognitive self-consciousness, yet the participant with the negative experience was more preoccupied with the AVH and had stronger beliefs regarding the need to control thoughts and the uncontrollability of thoughts. However, the BAVQ-R showed reduction of experienced malevolence and omnipotence. Moreover, nonsignificant correlations were found in the direction of more positive content and control of voices across the days of app use, as shown in Table 2.

### Privacy Concerns

The participants reported concerns about personal privacy when using electronic devices for symptom assessment and considered it positive that the iPod was offline during the pilot study. This is in accordance with a study showing that one-third of a sample of young adults with psychotic disorders experienced discomfort in online settings [37]. This finding suggests that personal privacy concerns should be carefully discussed before data sampling is initiated. It also suggests that offline sampling devices, such as iPods, are perhaps preferred if the participants are uncomfortable using online apps.

### Adherence to the Protocol

Previous mobile phone studies including adolescents and adults with psychotic disorders showed enrollment and dropout rates of approximately 50% and 5%, respectively [25,26]. However, comparisons of enrollment and dropout rates in this study are difficult due to the small number of participants included. The two participants compliant with the sampling procedure had a mean response rate of 74% over an average of 12 days, which is considerably more than the recommended minimum of 33% [48]. This finding indicates that adolescents with EOP are willing to participate in research using electronic devices to self-assess symptoms several times per day. The mean response rate also illustrates that the participants confirmed having AVH several times per day for a minimum 1 week, which is consistent with the moderate hallucination score they received on the PANSS and suggests that the app measured the target construct of hallucinations. According to the noncompliant participant, the insufficient amount of sampling was primarily caused by having to bring two devices to daily activities. This suggests that the noncompliance was a result of the pilot test (having to bring two devices), not the app itself.

The number and randomness of notifications and the repetitiveness of the statements were reported as too much and associated with discomfort. Studies have found that too many notifications and repetitive self-assessments may result in higher dropout rates in ESM studies and a biased representation of participants completing the sampling period [38,48]. Furthermore, although random notifications are preferred in ESM studies [48], this was associated with discomfort among the participants in this pilot study, suggesting that compliance among adolescents with EOP may increase by having fewer daily notifications than in studies involving adult patients, and having notifications at fixed time points instead of at random times. Alternatively, the use of machine learning approaches has been suggested as a means to individualize the number of notifications and variation in questions, and thus increase compliance and engagement with ESM studies [36,57].

### Integration into Clinical Care

One participant considered integrating technology into clinical care as a potentially useful alternative to verbal communication if the voices were not in control. However, the interpersonal aspect of existing care was emphasized as an important factor. This finding is in accordance with a previous study in adults with psychotic disorders, showing that technology may be perceived as a threat to existing care and that self-reports may be biased [36]. Furthermore, although support was offered during the sampling period, we were not able to capture the mixed experiences of the participants over the phone. In a mobile phone study of adults with psychotic disorders, Kumar and colleagues [26] found lower completion rates among participants with more severe symptoms, suggesting that additional support is warranted in such cases. In sum, these findings suggest that the use of mobile phones as part of clinical care is viable for adolescents with EOP as an addition to existing care. The results also suggest that the assessment of psychotic symptoms may be more suitable for use over shorter time periods, (eg, after discharge from the hospital, starting a new medication), as suggested by Palmier-Claus and colleagues [36], and that adolescents may require more support during the sampling period compared to adults.

### Conclusions

This study shows that including patients’ subjective experiences in the development and pilot-testing of health care technology may provide useful information in addition to statistical analyses. Based on the mean response rate of the two participants included in the statistical analyses, the use of mobile phones with ESM in adolescents with EOP seems feasible, although this warrants further investigation with larger samples. Regarding acceptability, there are limitations with the use of apps, and care should be taken such that the app is perceived as meaningful, comfortable, and safe. As mentioned previously, limitations may be that apps produce unexpected distressing thoughts and unwanted focus on negative feelings such as anxiety. Therefore, it may help to have a plan if distress is experienced. As an alternative to online apps, offline versions could be considered to avoid the risk of inducing or reinforcing unwanted persecutory delusions. Our conclusion is that the use of app technology for real-time monitoring of psychotic symptoms can provide new knowledge of daily and even hourly fluctuations and severity of hallucinatory episodes, which can increase diagnostic resolution. Moreover, such information can have therapeutic effects if it corresponds with similar fluctuations in cognitive control and experienced distress, for example, which can be suitable targets for psychosocial interventions, such as cognitive therapy.

## Abbreviations

AVH: auditory verbal hallucinations
BAVQ-R: Beliefs About Voices Questionnaire-revised version
CGAS: Children’s Global Assessment Scale
EOP: early-onset psychosis
ESM: experience sampling method
IQ: intelligence quotient
PANSS: Positive and Negative Syndrome Scale
VAS: visual analog scale
